# Ginsenoside Rb1 and Rd Remarkably Inhibited the Hepatic Uptake of Ophiopogonin D in Shenmai Injection Mediated by OATPs/oatps

**DOI:** 10.3389/fphar.2018.00957

**Published:** 2018-08-22

**Authors:** Xiaopei Liu, Lin Chen, Mingyi Liu, Hong Zhang, Shibo Huang, Yuqing Xiong, Chunhua Xia

**Affiliations:** ^1^Clinical Pharmacology Institute, Nanchang University, Nanchang, China; ^2^The Second Hospital of Anhui Medical University, Hefei, China; ^3^Nanchang Hongdu Hospital of TCM, Nanchang, China

**Keywords:** Shenmai injection, ophiopogonin D, OATPs, compatibility mechanism, hepatic uptake

## Abstract

Shenmai injection (SMI) is derived from traditional Chinese herbal prescription Shendong yin and widely used for treating cardiovascular diseases. Ophiopogonin D (OPD) is one of the main active components of SMI. The hepatic uptake of OPD is mediated by organic anion-transporting polypeptides (OATPs/oatps) and inhibited by some other components in SMI. This study aimed to identify the active components of SMI responsible for the inhibitory effects on hepatic uptake of OPD in rats and explore the compatibility mechanisms of complex components in SMI based on OATPs/oatps. The known effective fractions, the known components in Shenmai Formula, and the fractions obtained from SMI by HPLC gradual-separation technology were individually/combinedly tested for their effects on OPD uptake in rat primary hepatocytes and recombinant OATP1B1/OATP1B3-expressing HEK293T cells. The results indicated that the OPD uptake was inhibited by panaxadiol-type ginsenosides (ginsenoside Rb1 and Rd), but slightly influenced by panaxatriol-type ginsenosides in rat primary hepatocytes and recombinant cells. The fractions of SMI-3-1 (0–11 min) and SMI-3-3 (15–20 min) obtained by HPLC gradual-separation technology were proved to be the major effective fractions that influenced the OPD uptake, and subsequently identified as ginsenoside Rb1 and Rd, respectively. The plasma concentrations of OPD in rats given OPD+ginsenoside Rb1+ginsenoside Rd were higher compared to rats given OPD alone at the same dose. In conclusion, ginsenoside Rb1 and Rd are the major effective components in SMI that remarkably inhibited the hepatic OPD uptake mediated by OATPs/oatps. The interaction of complex components by OATPs/oatps may be one of the important compatibility mechanisms in SMI.

## Introduction

Traditional Chinese medicine is the quintessence of the cultural heritage of the Chinese nation as well as the world. In order to reach the goals of better curative efficacy and fewer side effects, multi-herb therapy has been utilized for thousands of years in China and other countries. It is an essential component of traditional medicine systems. In TCM, the compatibility of herbs in long-term clinical practices showed its significance, and several inspiring experiences have been accumulated (Yi and Chang, 2004). Compatibility refers to the combination of two or more herbs based on the clinical settings and the properties of herbs (Tang, 2003). Compatibility mechanism of TCM is still an urgent and vital issue that needs to be elucidated to realize its modernization (Wang et al., 2012). Previous studies on compatibility mechanisms of TCM mainly focused on the efficacy when two or more herbs were co-administered. But recent studies on herb compatibility concentrated on the variations of chemical components (Xu et al., 2015), pharmacological effects (Asl and Hosseinzadeh, 2008; Orazizadeh et al., 2014), and pharmacokinetic characteristics that refer to metabolizing enzymes (Liu et al., 2013; Tai et al., 2014).

Moreover, transporters play a vital role in the *in vivo* process of absorption, distribution, and excretion of drugs. OATPs/oatps are a kind of important membrane uptake transport proteins in humans as well as animals. In human liver, OATP1B1 and OATP1B3 are the two most important and extensively studied transporters. These are exclusively expressed in the basolateral membrane of the liver and are responsible for the uptake of a wide variety of endogenous and exogenous substrates, such as antidiabetics, antineoplastics, HMG-CoA reductase inhibitors and so on (Klatt et al., 2013; Van De Steeg et al., 2013; Kunze et al., 2014; Marada et al., 2015). Furthermore, several studies have shown that many herbal ingredients may impact the transportation of substrates mediated by OATP1B1/1B3 (Mandery et al., 2012; Zhang et al., 2013; He et al., 2015; Sheng et al., 2015). The interactions among these herbal ingredients based on OATPs might be one of the important compatibility mechanisms of TCM.

Shenmai injection (SMI) is derived from the famous traditional Chinese herbal prescription Shendong yin, whose formulation was first recorded in *Zhengyin Maizhi* by Jing-Ming Qin in 1702 AD (Lu et al., 2014). According to the theory of TCM, Shendong yin consists of ginseng and ophiopogon japonicas, which invigorates the Qi for relieving desertion and nourishes the Yin for replenishing bodily fluids (Hu et al., 2012). SMI is an extract of ginseng and ophiopogon japonicas, and is now widely used in China for the treatment of heart failure, shock, senile cerebral infarction, and so on. Ginsenosides and ophiopogonis have been isolated from ginseng and ophiopogon japonicas, and are regarded as the principal constituents responsible for the pharmacological activities (Ye et al., 2015). The former mainly consists of PDG (including ginsenoside Rb1, ginsenoside Rd, etc.), PTG (including ginsenoside Re, ginsenoside Rg1, etc.) and oleanolic acid (ginsenoside Ro), and the latter mainly refers to OPD (Zhan et al., 2015).

Our previous study demonstrated that the plasma concentrations of OPD in rats administered with SMI are higher than those administered with the same dose of OPD, and the ratio of the plasma concentration to the liver tissue concentration of OPD in SMI group was about 5–7 times greater than those in OPD group (Xia et al., 2008a). We also found that OPD can be taken by rat primary hepatocytes, and transported by OATP1B1-expressing HEK293 cells (Zhang et al., 2017). These results indicated potential interactions between other components and OPD in SMI treated group.

Therefore, the purpose of the present study was to identify the active components of SMI that are responsible for the inhibitory effects on hepatic uptake of OPD in rats and explore the compatibility mechanism of complex components in SMI mediated by OATPs/oatps.

## Materials and Methods

### Chemicals and Reagents

Shenmai injection (50 mL) was provided by Hebei Shenwei Pharmaceutical Co., Ltd. According to LC-MS method, the content of SMI included ginsenoside Rg1 (81.01 μg/mL), ginsenoside Re (68.78 μg/mL), ginsenoside Rd (78.24 μg/mL), ginsenoside Rb1 (198.30 μg/mL) and OPD (12.44 μg/mL), respectively, as described previously (Xia et al., 2008b) (Lot Number: 130427312). OPD (purity > 98%) was purchased from Shanghai Yiji Industrial Co., Ltd. (Shanghai, China) (Lot Number: 14082001). Ginseng total saponins (purity > 80%, containing ginsenoside Rb1 272.39 μg, ginsenoside Rd 197.61 μg, ginsenoside Re 125.75 μg and ginsenoside Rg1 98.12 μg per milligram) (Lot Number: 20140513), PDG (purity > 80%, containing ginsenoside Rb1 416.82 μg and ginsenoside Rd 237.93 μg per milligram) (Lot Number: 20140515), PTG (purity > 80%, containing ginsenoside Re 334.32 μg and ginsenoside Rg1 129.29 μg per milligram) (Lot Number: 2014 0430), ginsenoside Rb1 (purity > 98%, Lot Number: 20140603), ginsenoside Rd (purity > 98%, Lot Number: 20140712), ginsenoside Re (purity > 98%, Lot Number: 20140607), and ginsenoside Rg1 (purity > 98%, Lot Number: 20140820) were all obtained from Tianjin Shilan Biotechnology Co., Ltd. (Tianjin, China). Digoxin was purchased from the Control of Pharmaceutical and Biological Products of Shanghai (Shanghai, China) (Lot Number: 100015-299709). Dimethyl sulfoxide (DMSO) was purchased from AMRESCO Inc. (United States). Methanol, acetonitrile and ethyl acetate were all of HPLC grade and purchased from Merck (Darmstadt, Germany). All other chemicals used in the study were of analytical grade and commercially available.

### Animals

Sprague–Dawley rats (180–240 g) were purchased from the Animal Experiment Center of Nanchang University. All rats were kept in a standard animal laboratory under controlled environmental conditions of temperature and humidity (20–22°C, 45 ± 5%), and 12/12h light-dark cycle. These animals had free access to water and chow diet, but were fasted for 12 h before initiating the experiments. The experimental protocols were approved by the Animal Ethics Committee of Nanchang University (NDSYDWLI-201512) and were performed in accordance with the guidelines of the National Institutes of Health for the Care and Use of Laboratory Animals.

### Uptake Study Using Isolated Rat Primary Hepatocytes

The known effective fractions and the known components in Shenmai Formula were individually/combinedly tested for their effects on OPD uptake in rat primary hepatocytes (all the compounds were dissolved in DMSO, and the final concentration of DMSO in uptake buffer was less than 0.5%). Hepatocytes were isolated from male Sprague–Dawley rats using EDTA-PBS and collagenase by *in situ* perfusion method (Maeda and Sugiyama, 2010). After isolation, cells were filtered through 200 mesh screen, centrifuged for 4 min at a speed of 600 rpm at 4°C, and then re-suspended in DMEM (Gibco, United States) containing 10% fetal calf serum. Cell viability was determined by trypan blue exclusion test and always exceeded 85%. Hepatocytes at a density of 1 × 10^6^/mL were seeded into 12-well plates and pre-warmed at 37°C for 10 min before the uptake experiment, which was initiated by supplementing with an additional 1mL of uptake buffer containing OPD (4 μM) and a series of concentrations of perpetrators. The series of concentrations of perpetrators, SMI, GTS, PDG, PDG+PTG, ginsenoside Rb1, ginsenoside Rb1+ginsenoside Rd, and ginsenoside Rb1+ginsenoside Rd+ginsenoside Re+ginsenoside Rg1 were diluted to 15.625, 31.25, 62.5, 125, 250, 500, and 1000 nM with uptake buffer in terms of ginsenoside Rb1; PTG, ginsenoside Re and ginsenoside Re+ginsenoside Rg1 were diluted to 6.25, 12.5, 25, 50, 100, 200, and 400 nM with uptake buffer in terms of ginsenoside Re; ginsenoside Rd was diluted to 6.25, 12.5, 25, 50, 100, 200, and 400 nM, and ginsenoside Rg1 was diluted to 7.8125, 15.625, 31.25, 62.5, 250, and 500 nM with uptake buffer. Control cells treated with an equal volume of uptake buffer containing 0.5% DMSO were used as a vehicle for treatment. After incubating for 10 min at 37°C, the cell suspensions were transferred into 1.5-mL plastic tubes and centrifuged for 4 min at a speed of 600 rpm, and then the supernatants were aspirated to terminate the uptake. Subsequently, the cell pellets were washed thrice with PBS at 4°C as described in our previous study (Zhang et al., 2017). Cells were then lysed with 1 mL of sterilized ultrapure water by repeated freezing and thawing. Of which, 200 μL was used for determining OPD concentration by using the LC-MS method as described previously and 50 μL was used to determine the protein content using the BCA assay (Zhang et al., 2017).

### Fractions of SMI Separated Using an HPLC System

The HPLC separation was performed using a Shimadzu HPLC-2010A (Shimadzu, Japan) with a Phenomenex^®^ Genmini C_18_ column (250 mm × 4.6 mm, 5 μm). The mobile phase consisted of solvent A (water) and solvent B (acetonitrile). A gradient elution program used was as follows: 0–10 min, 10→20% (B); 10–30 min, 20→20% (B); 30–60 min, 20→40% (B); 60–90 min, 40→85% (B); 90–100 min, 85→10% (B); 100–110 min, 10→10% (B) (condition 1) (Zeng et al., 2013). The flow rate was set at 1 mL/min, and the column was run at 40°C. The eluate was monitored by an ultraviolet detector at 203nm, and four fractions were collected based on the HPLC chromatogram and the retention times of the peaks as follows: SMI-1 (0–30 min), SMI-2 (30–45 min), SMI-3 (45–62 min), and SMI-4 (62–100 min). Then, each fraction was evaporated to dryness under nitrogen stream at 60°C, and each residue was dissolved in 200 μL methanol or DMSO and reserved for subsequent HPLC qualitative analysis or uptake study (final concentration of DMSO less than 0.5%), respectively.

The SMI-3 fraction (45–62 min) eluted under the above HPLC conditions (condition 1) was further separated by another HPLC condition (condition 2). The separation was performed on a Synergi C_18_ column (250 mm × 4.6mm, 4 μm) with a mobile phase of solvent A (water) and solvent B (acetonitrile). A gradient elution used was as follows: 0–20 min, 35→40% (B); 20–21 min, 40→30% (B); 21–31 min, 30→30% (B) (condition 2). By using this HPLC separation, the following fractions were collected: SMI-3-1 (0–11 min), SMI-3-2 (11–15 min), and SMI-3-3 (15–20 min). Likewise, each fraction was evaporated to dryness and then dissolved in 200 μL methanol or DMSO for subsequent HPLC qualitative analysis or uptake study, respectively.

### Uptake Study Using Transporter Expression Systems

To investigate the relationship between OPD and OATP1B3, the time-dependent and concentration-dependent uptake experiments of OPD in HEK293T cells that stably express OATP1B3 (provided by Shanghai Genechem Co., Ltd., Shanghai, China) were conducted. OATP1B3-transfected HEK293T cells were seeded in 12-well plates at a density of 1.5 × 10^5^ cells/well, and 1 mM sodium butyrate was added to the cells to induce protein expression when they reached confluence. After 24 h, the medium was removed, and the cells were washed with pre-warmed (37°C) uptake buffer, and then pre-incubated with 1mL pre-warmed uptake buffer for 20 min. This was followed by the initiation of uptake by supplementing an additional 1mL uptake buffer containing 8 μM OPD, incubation for 5, 10,15, 20, 30, 40, 60 min at 37°C, and termination of the incubation by removing the uptake solution and washing the cells thrice with 1mL of ice-cold uptake buffer. Cells were then lysed with 1mL of sterilized ultrapure water by freezing and thawing repeatedly. Intracellular accumulation of OPD was measured using LC-MS method as described in our previous paper. The protein concentration of each sample was determined using the BCA Protein Assay (Zhang et al., 2017). Likewise, the concentration-dependent experiments were initiated by supplementing an additional 1mL uptake buffer that contains a series of concentrations of OPD (1, 2, 4, 8, 16, 32, 64, and 100 μM). This was followed by an incubation of 10 min, and the remaining procedures were similar to the time-dependent experiments.

Subsequently, the influences of SMI, GTS, known effective fractions (PDG, PTG) and their assemblage (PDG+PTG), known effective components and their assemblages (ginsenoside Rb1, ginsenoside Rd, ginsenoside Rb1+ginsenoside Rd, ginsenoside Re, ginsenoside Rg1, ginsenoside Re+ginsenoside Rg1, and ginsenoside Rb1+ginsenoside Rd+ginsenoside Re+ginsenoside Rg1), preliminarily separated fractions and their assemblages (SMI-1, SMI-2, SMI-3, SMI-4, SMI-1+SMI-2+ SMI-4, and SMI-1+SMI-2+SMI-3+SMI-4), and secondary separated fractions (SMI-3-1, SMI-3-2, and SMI-3-3) on the uptake of OPD were investigated in HEK293T cells that stably expressed OATP1B1 or OATP1B3. Before the transporting assay, the cells were seeded at a density of 1.5 × 10^5^ cells/well and induced by 1 mM sodium butyrate for 24 h. After pre-incubating with uptake buffer at 37°C for designated time, the uptake studies were initiated by adding the uptake buffer containing OPD (8 μM) and a range of concentrations of the perpetrator for designated time and terminated with ice-cold uptake buffer. The concentration of OPD was determined by the LC-MS method as described previously (Zhang et al., 2017).

The series of concentrations of SMI, GTS, known effective fractions, known effective components and their assemblages were set according to the uptake study in rat primary hepatocytes. While the HPLC separated fractions of SMI-3, SMI-1+ SMI-2+ SMI-3+ SMI-4, and SMI-3-1 were diluted to 15.625, 31.25, 62.5, 125, 250, 500, 1000 nM with uptake buffer in terms of ginsenoside Rb1; SMI-2, SMI-1+ SMI-2+ SMI-4 were diluted to 6.25, 12.5, 25, 50, 100, 200, 400 nM in terms of ginsenoside Re; SMI-3-3 were diluted to 6.25, 12.5, 25, 50, 100, 200, 400 nM in terms of ginsenoside Rd; SMI-1, SMI-4 and SMI-3-2 could not be quantified exactly and thereby diluted one by one.

### Effect of Identified Active Components on the Pharmacokinetics of OPD in Rats *in vivo*

Twelve Sprague–Dawley rats were randomly divided into two groups, with each group containing 3 males and 3 females. The weight of the rats in the two groups was comparable (group A: 213.0 ± 14.9 g and group B: 214.7 ± 14.2 g). After intravenous administration of OPD at a dosage of 77.0 μg/kg (group A) or the mixture of ginsenoside Rb1+ginsenoside Rd+OPD (928 μg/kg+314 μg/kg+77.0 μg/kg, respectively, group B) via the tail vein, blood samples (about 200 μL) were drawn through orbital vein and collected into heparinized polythene tubes at 0, 2, 10, 20, 30, 45, 60, 90, 120, and 180 min and immediately centrifuged at 3000 rpm for 5 min. The plasma samples obtained were then stored at −80 °C and the concentrations of OPD were analyzed by the following LC-MS/MS method.

### Quantification of OPD in Rat Plasma by LC-MS/MS

LC-MS/MS was performed using Agilent 6430A LC-MS/MS system coupled with an Agilent 1200 series HPLC system (Agilent, United States). Chromatographic separation was achieved on a Shimadzu Pack VP-ODS C_18_ (150mm × 2.0 mm, 5 μm) column, with the mobile phase of 0.02% acetic acid (A) and acetonitrile (B). The gradient elution program used was as follows: 0–3.0 min, 45→90% (B); 3.0–3.5 min, 90→90% (B); 3.5–3.6 min, 90→45% (B); 3.6–6.0 min, 45→45% (B), and the flow rate was set at 0.2 mL/min. The analysis was carried out using a selected ion monitoring (SIM) mode at m/z 853.50 for OPD and 779.40 for digoxin (internal standard). The ESI source was utilized, and the operating parameters of the MS were set as below: gas flow, 8.0 L/min; gas temperature, 350°C; capillary voltage, −4000 V; nebulizer pressure, 40psi.

An aliquot of 10 μL digoxin (400 ng/mL) as the internal standard was added to 100 μL plasma sample in a 1.5-mL plastic test tube. The mixture was vortexed for 30 s and extracted with 1000 μL of ethyl acetate. After vortex-mixing for 5 min and centrifuging at 15000rpm for 10 min, the upper organic layer was quantitatively transferred to another tube and evaporated to dryness using a vacuum evaporator (Thermo scientific, United States) at 45°C. Then, the residue was reconstituted in 100 μL methanol and centrifuged at 20000 rpm for 5 min. A 50 μL aliquot of the supernatant was injected into the LC-MS/MS system for analysis.

### Data Analysis

All experiments were performed in triplicate. The mean values were presented as standard deviations (mean ± SD). SPSS v21.0 software was used to perform ANOVA for comparing the experimental groups with the control group and calculated the IC_50_ values. The pharmacokinetic parameters in rats *in vivo* were obtained by DAS 2.0, and unpaired Student’s *t*-test was carried out to acquire the differences between group A and group B. *p* < 0.05 was considered to be statistically significant.

## Results

### Impact of the Known Effective Fractions and the Known Effective Components in Shenmai Formula on Hepatic Uptake of OPD in Rat Primary Hepatocytes

**FIGURE 1:**
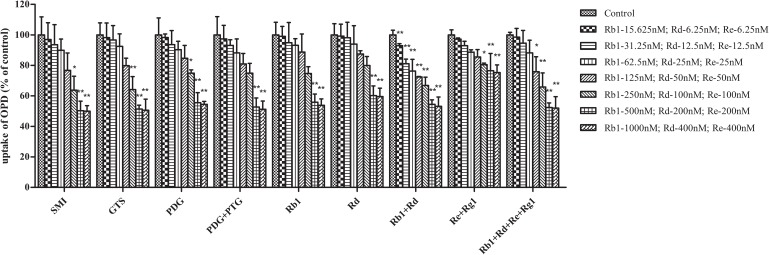
Inhibitory effects of the known effective fractions and the known effective components in Shenmai Formula on hepatic uptake of OPD (4 μM) in rat primary hepatocytes. The series of concentrations of SMI, GTS, PDG, PDG+PTG, Rb1+Rd, and Rb1+Rd+Re+Rg1 were presented in terms of that of Rb1, Re+Rg1 in terms of Re. The uptake of OPD in rat primary hepatocytes was described as the percentage of the control and each point represented mean ± SD of three consecutive experiments. “^∗^” represents *p* < 0.05, “^∗∗^” represents *p* < 0.01 compared to the control group.

As shown in **Figure 1**, SMI demonstrated concentration-dependent inhibitory effects on the uptake of OPD in rat primary hepatocytes. The IC_50_ value calculated by SPSS software v21.0 was 0.66 μM (in terms of ginsenoside Rb1 content). Furthermore, the influence of the known effective fractions and the known effective components were also investigated. The results demonstrated that the uptake of OPD in rat primary hepatocytes could be inhibited by GTS, PDG, mixture of PDG+PTG, ginsenoside Rb1, ginsenoside Rd, mixture of ginsenoside Rb1+ ginsenoside Rd, and mixture of ginsenoside Rb1+ ginsenoside Rd+ ginsenoside Re+ ginsenoside Rg1 with IC_50_ values of 0.67, 0.94, 0.83, 0.89, 0.44, 0.87, and 0.71 μM (in terms of ginsenoside Rb1 content except ginsenoside Rd group), respectively (**Table 1**). While the high concentrations of PTG, ginsenoside Re, ginsenoside Rg1 and the mixture of ginsenoside Re+ginsenoside Rg1 slightly affected the uptake of OPD compared with the control group. These results suggested that PDG may be the major effective fraction that exerts impact on the uptake of OPD, while ginsenoside Rb1 and ginsenoside Rd were the potentially effective components that accounted for these results in rat primary hepatocytes.

**Table 1:** Half-maximal inhibitory concentrations (IC_50_) (μM) of the known effective fractions, the known effective components and the HPLC separation fractions in SMI on the uptake of OPD in rat primary hepatocytes, OATP1B1- and OATP1B3-HEK293T cells.

	Rat primary hepatocytes	OATP1B1-HEK293T cells	OATP1B3-HEK293T cells
SMI	0.66	1.28	0.52
GTS	0.67	1.19	0.35
PDG	0.94	1.13	0.46
PTG	INVALD	INVALD	2.44
PDG+PTG	0.83	1.39	0.43
Rb1	0.89	1.64	0.57
Rd	0.44	1.98	0.32
Rb1+Rd	0.87	1.52	0.5
Rg1	INVALD	INVALD	4.18
Re	INVALD	INVALD	INVALD
Rg1+Re	INVALD	INVALD	3.79
Rb1+Rd+Rg1+Re	0.71	1.04	0.45
SMI-1	N.D	INVALD	INVALD
SMI-2	N.D	INVALD	3.39
SMI-3	N.D	1.48	0.5
SMI-4	N.D	INVALD	INVALD
SMI-1+2+4	N.D	INVALD	2.65
SMI-1+2+3+4	N.D	1.33	0.48
SMI-3-1	N.D	1.94	0.55
SMI-3-2	N.D	INVALD	INVALD
SMI-3-3	N.D	1.66	0.31

### Impact of the Known Effective Fractions and the Known Effective Components in Shenmai Formula on Hepatic Uptake of OPD in Transporter Expression Systems

The results of uptake kinetic study in OATP1B3-transfected HEK293T cells indicated that the whole uptake process of OPD for OATP1B3 followed a typical Michaelis-Menten equation, and the parameters K_m_ and V_max_ were calculated to be 11.27 μM and 146.5 pmol/min/mg protein via GraphPad Prism v5.0, respectively. In addition to K_m_ of 5.50 μM in OATP1B1-HEK293T cells (Zhang et al., 2017), 8 μM was selected as the concentration of the OPD substrate for subsequent experiments.

**FIGURE 2:**
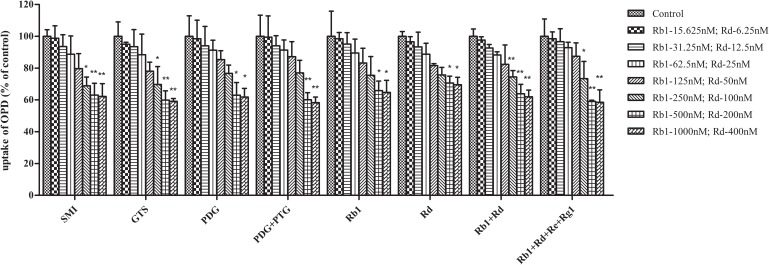
Inhibitory effects of the known effective fractions and the known effective components in Shenmai Formula on OATP1B1-mediated uptake of OPD (8 μM). The series of concentrations of SMI, GTS, PDG, PDG+PTG, Rb1+Rd, and Rb1+Rd+Re+Rg1 were presented in terms of that of Rb1. The uptake of OPD was calculated by subtracting the uptake in control cells from that in transporter-expressing cells, and results were shown as the percentage of the control (mean ± SD, *n* = 3). “^∗^” represents *p* < 0.05, “^∗∗^” represents *p* < 0.01 compared to the control group.

**FIGURE 3:**
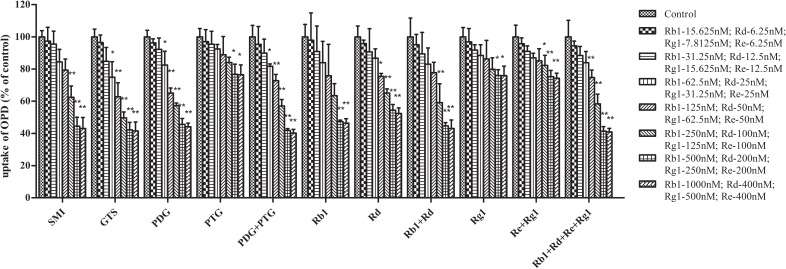
Inhibitory effects of the known effective fractions and the known effective components in Shenmai Formula on OATP1B3-mediated uptake of OPD (8 μM). The series of concentrations of SMI, GTS, PDG, PDG+PTG, Rb1+Rd, and Rb1+Rd+Re+Rg1 were presented in terms of that of Rb1, PTG, and Re+Rg1 in terms of Re. The uptake of OPD was calculated by subtracting the uptake in control cells from that in transporter-expressing cells, and results were shown as the percentage of uptake of the control (mean ± SD, *n* = 3). “^∗^” represents *p* < 0.05, “^∗∗^” represents *p* < *0.01* compared to the control group.

Moreover, the impact of the known effective fractions and the known effective components in Shenmai Formula on hepatic uptake of OPD in transporter expression systems were explored (**Figures 2**, **3**). Similar results were obtained compared to those in rat primary hepatocytes. SMI could inhibit the uptake of OPD in both OATP1B1- and OATP1B3-HEK293T cells with IC_50_ values of 1.28 and 0.52 μM (in terms of ginsenoside Rb1 content), respectively. The uptake of OPD in OATP1B1-HEK293T cells was inhibited by GTS, PDG, mixture of PDG+PTG, ginsenoside Rb1, ginsenoside Rd, mixture of ginsenoside Rb1+ginsenoside Rd, and mixture of ginsenoside Rb1+ginsenoside Rd+ginsenoside Re+ginsenoside Rg1 (in terms of ginsenoside Rb1 content except ginsenoside Rd group) with IC_50_ values of 1.19, 1.13, 1.39, 1.64, 1.98, 1.52 and 1.04 μM, respectively. When shifting the scenario to OATP1B3-HEK293T cells, GTS, PDG, and mixture of PDG+PTG significantly reduced the uptake of OPD, and the IC_50_ values were calculated to be 0.35, 0.46, and 0.43 μM, respectively. The known effective components, ginsenoside Rb1, ginsenoside Rd, mixture of ginsenoside Rb1+ginsenoside Rd, and ginsenoside Rb1+ginsenoside Rd+ginsenoside Re+ginsenoside Rg1 decreased the uptake of OPD with IC_50_ values of 0.57, 0.32, 0.50, and 0.45 μM, respectively (**Table 1**). But PDG and ginsenoside Rg1 showed weak inhibitory effects on the uptake of OPD. The results acquired in the transporter expression systems also indicated that PDG may be the major effective fraction, while ginsenoside Rb1 and ginsenoside Rd were the effective components that exert impact on the uptake of OPD.

### Impact of HPLC Gradual-Separation Fractions of SMI on Hepatic Uptake of OPD Mediated by OATPs

**FIGURE 4:**
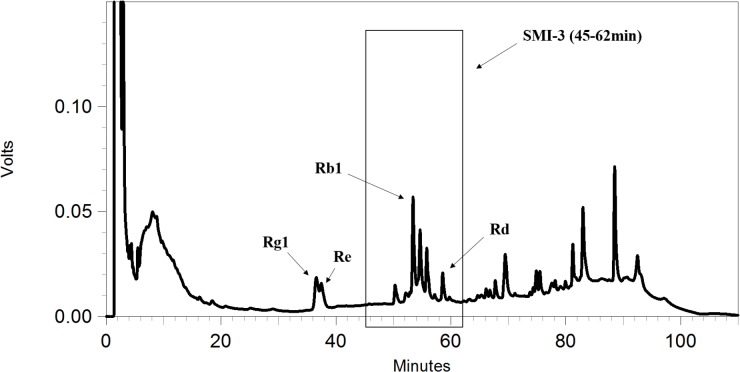
HPLC chromatograms of SMI obtained under condition 1.

**FIGURE 5:**
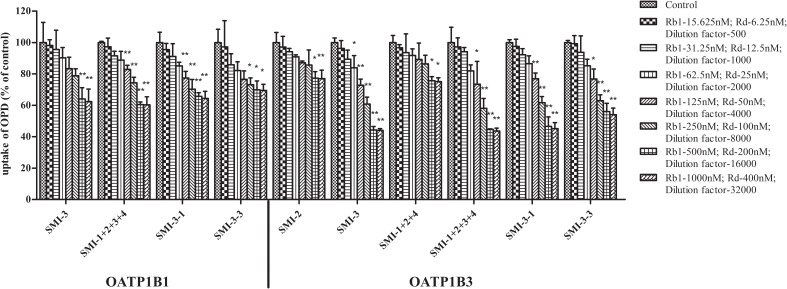
Inhibitory effects of the preliminarily separated fractions and the secondary separated fractions of SMI obtained by HPLC on the OATP1B1/1B3 mediated uptake of OPD (8 μM). The series of concentrations of SMI-3, SMI-1+2+3+4, and SMI-3-1 were presented in terms of that of Rb1, SMI-3-3 in terms of Re. SMI-2 and SMI-1+2+4 in terms of dilution factors. The uptake of OPD was calculated by subtracting the uptake in control cells from that in transporter-expressing cells, and the results were shown as the percentage of the control (mean ± SD, *n* = 3). “^∗^” represents *p* < 0.05, “^∗∗^” represents *p* < 0.01 compared to the control group.

**FIGURE 6:**
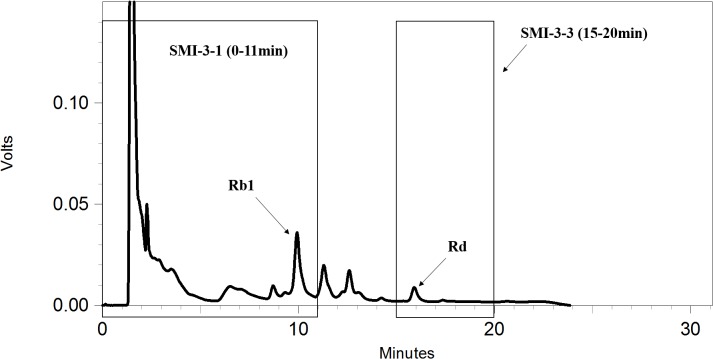
HPLC chromatograms of SMI-3 (45–62 min) obtained under condition 2.

To further identify the components in SMI that are responsible for the inhibition of OPD uptake mediated by OATP1B1/1B3, an HPLC gradual-separation technology was used in our study. A representative HPLC chromatogram of SMI was shown in **Figure 4**. Four separated fractions were collected based on the HPLC chromatogram and the retention times of the peaks. The effect of each separated fraction on the uptake of OPD mediated by OATP1B1/1B3 was investigated. Of these four fractions, only fraction SMI-3 (45–62 min) or the mixture of SMI-1+SMI-2+SMI-3+SMI-4 showed marked inhibitory effects on the uptake of OPD in OATP1B1-HEK293T cells with IC_50_ values of 1.48 and 1.33 μM, respectively (**Figure 5** and **Table 1**). Similarly, in OATP1B3-HEK293T cells, fraction SMI-3 (45–62 min) and the mixture of SMI-1+ SMI-2+ SMI-3+ SMI-4 demonstrated significant inhibitory effects on the uptake of OPD with the IC_50_ values of 0.50 and 0.48 μM, respectively. Subsequently, fraction SMI-3 (45–62 min) was further separated using another HPLC condition (condition 2). As shown in **Figure 6**, several peaks were identified in fraction SMI-3 (45–62 min). Of these, the peak with retention time of 10 min from fraction SMI-3-1 (0–11 min) and the peak with retention time of 16 min from fraction SMI-3-3 (15–20 min) were identified as Rb1 and Rd, respectively, by comparing the chromatographic behaviors of these two fractions with the standards of ginsenoside Rb1 and ginsenoside Rd. The two fractions SMI-3-1 (0–11 min) and SMI-3-3 (15–20 min) demonstrated significant inhibitory effects on the uptake of OPD with respective IC_50_ values of 1.94 and 1.66 μM for OATP1B1, 0.55 and 0.31 μM for OATP1B3 (**Figure 5** and **Table 1**). These results further confirmed that ginsenoside Rb1 and ginsenoside Rd were the main effective components of SMI that affected the hepatic uptake of OPD mediated by OATPs/oatps.

### Impact of the Responsible Components Ginsenoside Rb1 and Ginsenoside Rd on the Pharmacokinetics of OPD in Rats

**FIGURE 7:**
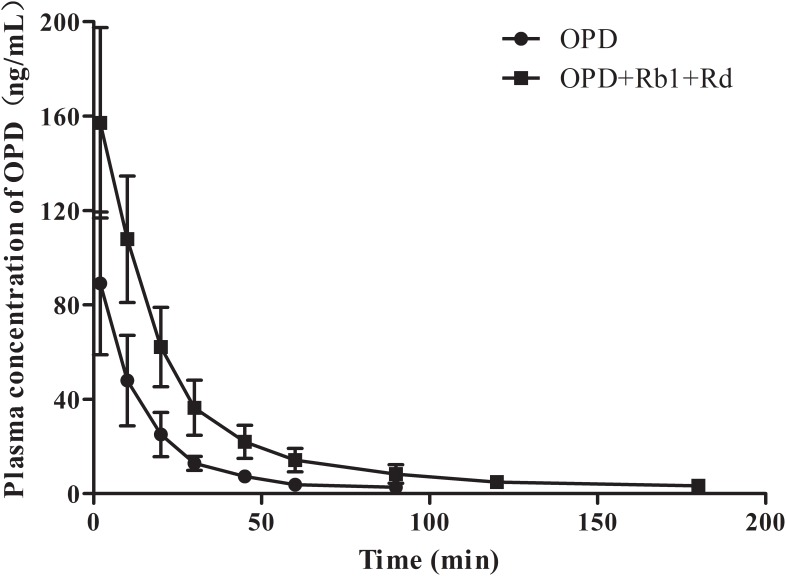
Mean plasma concentration-time curves of OPD in rats after intravenous administration of OPD and mixture of OPD+ginsenoside Rb1+ginsenoside Rd at the same dose of 77.0 μg/kg OPD (*n* = 6).

To further validate that ginsenoside Rb1 and ginsenoside Rd are the components of SMI responsible for the inhibitory effects on hepatic uptake of OPD, a comparative pharmacokinetic study was conducted in rats. As shown in **Figure 7**, the plasma concentrations of OPD in rats administered with the mixture of OPD+ginsenoside Rb1+ginsenoside Rd (group B) were higher than those given the same dose of OPD (group A). The main pharmacokinetic parameters of the two groups, such as AUC_0-t_, AUC_0−∞_, C_max_, CL, t_1/2_, and MRT showed significant differences (*p* < 0.05, **Table 2**).

**Table 2:** Main pharmacokinetic parameters of OPD in rats after intravenous administration of OPD (group A) and mixture of OPD+ginsenoside Rb1+ ginsenoside Rd (group B) at the same dose of 77.0 μg/kg OPD (*n* = 6).

Parameters	Group A	Group B
MRT_0−t_(min)	19.08 ± 1.53	27.89 ± 5.90^∗∗^
t_1/2_(min)	16.79 ± 1.23	34.27 ± 10.14^∗^
CL(L/min/kg)	0.051 ± 0.016	0.020 ± 0.06^∗∗^
C_max_(ng/ml)	89.10 ± 30.24	157.14 ± 40.35^∗^
AUC_0−t_(ng/ml^∗^min)	1579.17 ± 551.06	4102.49 ± 1325.19^∗∗^
AUC_0−∞_(ng/ml^∗^min)	1634.86 ± 523.30	4245.05 ± 1369.97^∗∗^
Mean Weight (g)	213.0 ± 14.9	214.7 ± 14.2
Normalized C_max_(ng/ml)	5.38 ± 1.58	9.47 ± 2.22^∗^
Normalized AUC_0−t_(ng/ml^∗^min)	95.07 ± 28.07	247.21 ± 75.13^∗∗^
Normalized AUC_0−∞_(ng/ml^∗^min)	98.57 ± 26.23	255.80 ± 77.74^∗∗^

*For the dose normalization, C_max_, AUC_0−t_ and AUC_0−∞_ were divided by the given dosing of OPD (μg), respectively. ^∗^p < 0.05, ^∗∗^p < 0.01 compared to group A, an unpaired Student’s t-test was used for the comparison.*

## Discussion

Traditional Chinese Medicine has been considered as one of the advanced medical sciences until the late 17th century because of its unique philosophy and treatment pattern (Yi and Chang, 2004). Many Chinese herbal formulae have been widely explored, and their effectiiveness is gradually proved by *in vivo* studies and clinical trials. Multi-herb therapy is one of the most important characteristics of TCM, and the compatibility mechanism of herbs is the core idea of TCM theory. From the perspective of modern science, the compatibility laws not only reflected the comprehensive effects of TCM, but also profoundly revealed the interactions among complex components in TCM mediated by metabolizing enzymes and transporters.

Shenmai injection (SMI) is derived from the famous traditional Chinese herbal prescription Shendong yin which is comprised of the famous herb pair ginseng and ophiopogonis japonicas. SMI is widely used in clinics and the main active ingredients included were PDG (including ginsenoside Rb1, ginsenoside Rd, etc.), and PTG (including ginsenoside Re, ginsenoside Rg1, etc.), and OPD (Ye et al., 2015; Zhan et al., 2015).

**FIGURE 8:**
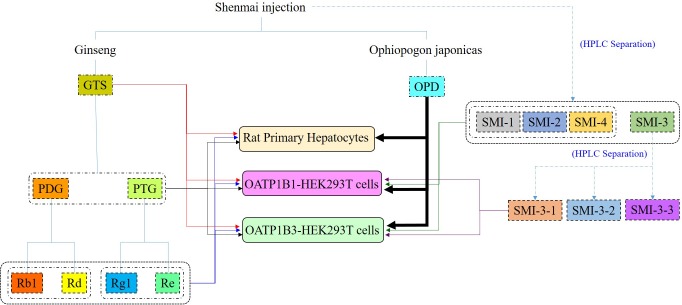
Study strategy for the compatibility mechanisms of SMI based on OATPs.

Results of our previous studies showed potential interactions between other constituents and OPD in SMI, leading to alterations in the magnitude of OPD transported from blood to hepatocytes (Xia et al., 2008a). Moreover, we also found that OPD could be taken by rat primary hepatocytes and transported by OATP1B1 (Zhang et al., 2017) and OATP1B3 (data shown in **Supplementary Materials**). To identify the potential components that accounted for the aforementioned results and explore the mechanisms behind the compatibility (the comprehensive strategy in present study shown in **Figure 8**), we disassembled Shenmai Formula into the known effective fractions (PDG and PTG) and known components (ginsenoside Rb1, ginsenoside Rd, ginsenoside Re and ginsenoside Rg1), and then assembled these fractions or components one by one into several formulas to individually measure their effects on the uptake of OPD in rat primary hepatocytes, OATP1B1- and OATP1B3-overexpressing HEK293T cells. The collective results showed that PDG may be the effective fraction, and ginsenoside Rb1 and ginsenoside Rd were the possible effective components of SMI that inhibited the uptake of OPD.

To further identify the possible active constituents, an HPLC gradual-separation technology was utilized to separate the complex components of SMI. The effluents were separated into four fractions (SMI-1: 0–30 min, SMI-2: 30–45 min, SMI-3: 45–62 min, SMI-4: 62–100 min) according to the HPLC chromatogram, and the effects of each fraction individually or in combination (SMI-1+ SMI-2+ SMI-4 and SMI-1+ SMI-2+ SMI-3+SMI-4) were investigated on the uptake of OPD in OATP1B1- and OATP1B3-overexpressing HEK293T cells. Both results indicated that there were some active components in the effluent SMI-3 (45–62 min), which resulted in the influence on the uptake of OPD mediated by OATP1B1 and OATP1B3. Therefore, SMI-3 (45–62 min) fraction was further separated using another HPLC condition until the main components were isolated as individual peaks on the HPLC chromatogram. SMI-3-1 (0–11 min) and SMI-3-3 (15–20 min) fractions displayed only one peak, and demonstrated significant inhibitory effects on the uptake of OPD mediated by OATP1B1 and OATP1B3. These were subsequently identified as ginsenoside Rb1 and ginsenoside Rd, respectively, by comparing the chromatographic behaviors of the two fractions with the standards of ginsenoside Rb1 and ginsenoside Rd.

To further validate the data, a comparative pharmacokinetic study in rats was performed. Twelve rats were randomly administered with OPD (77 μg/kg, group A) or the mixture of OPD +ginsenoside Rb1+ginsenoside Rd (77, 928, and 314 μg/kg, respectively, group B) and the doses were set in accordance with our previous study (Xia et al., 2008a). The results showed that the plasma concentrations of OPD in rats administered with the mixture (OPD + ginsenoside Rb1 + ginsenoside Rd) were higher than those given the same dose of OPD and the main pharmacokinetic parameters (AUC, C_max_, t_1/2_, CL, MRT and dose-normalized AUC, dose-normalized C_max_) between the two groups showed significant differences. The dose-normalized C_max_ and dose-normalized AUC_0-t_ of OPD in group B were 1.76-fold and 2.60-fold compared to group A, respectively. These results were nearly consistent with those obtained from our previous studies (Xia et al., 2008a). Based on the above results, it can be deduced that ginsenoside Rb1 and ginsenoside Rd inhibited the uptake of OPD from blood into hepatocytes mediated by OATPs/oatps, resulting in the increased exposure of OPD in blood circulation. This may be the underlying compatibility mechanism of complex components in Shenmai Formula.

The inhibition of ginsenoside Rb1 and Rd on OPD uptake in OATP1B1/1B3 transfected cells was compared with that in rat primary hepatocytes. The IC_50_ values obtained from rat primary hepatocytes were somewhere between that from OATP1B1-transfected cells and OATP1B3-transfected cells. This might be due to the comprehensive effects of transporters of homology with human transporters OATP1B1/1B3 in rat primary hepatocytes.

Tang et al. (2017) investigated the pharmacokinetics of the major components of Shenmai injection in 10 subjects with stable angina pectoris. The results showed maximal concentrations (C_max_) of 3466.04 ± 468.34 and 494.51 ± 110.24 μg/L, which were equal to 3.06 ± 0.41 and 0.51 ± 0.11 μM, respectively for ginsenoside Rb1 and Rd in humans. When comparing the C_max_ in humans with the obtained IC_50_ values in our study, the inhibitions of ginsenoside Rb1 and Rd on the uptake of OPD exhibited distinct clinical significance.

Why panaxadiol-type ginsenoside, ginsenoside Rb1 and ginsenoside Rd in SMI significantly inhibited the uptake of OPD in OATP1B1/1B3-HEK293T cells, but PTG, ginsenoside Re, and ginsenoside Rg1 only showed weak inhibitory effects? A recent study by Jiang et al. (2015) could provide support on the results obtained in the present study. Jiang et al. (2015) found that the active components, ginsenoside Rb1, and ginsenoside Rd, were not substrates of neither OATP1B1 nor OATP1B3, but exerted remarkable impact on the uptake of representative substrate estradiol-17β-glucuronide (E2-17β-G) in OATP1B1 and OATP1B3. While PTG, ginsenoside Re and ginsenoside Rg1 were substrates of OATP1B3, but showed weak inhibitory effects on the uptake of E2-17β-G. What about the underlying mechanisms? Since ginsenoside Re and ginsenoside Rg1 were substrates of OATP1B3, and does OPD influence their uptake into hepatocytes? These would be discussed in our upcoming study that elucidates the comprehensive mechanisms of compatibility in Shenmai Formula.

## Conclusion

In this study, a relatively comprehensive strategy to explore the underlying mechanisms of compatibility from the perspective of two major uptake transporters in liver has been established by dissembling and assembling the major effective fractions and the components in SMI via rat primary hepatocytes models and transporter expression systems. Also, the active fraction (PDG), and the active components (ginsenoside Rb1 and ginsenoside Rd) responsible for the inhibitory effects on the hepatic uptake of OPD were identified. The interaction among complex components in SMI mediated by OATPs/oatps may be one of the important compatibility mechanisms of TCM.

## Author Contributions

CX and YX participated in research design. XL, LC, ML, HZ, and SH conducted the experiments. XL, LC, and CX performed the data analysis. XL, LC, and CX wrote or contributed to the writing of the manuscript.

## Conflict of Interest Statement

The authors declare that the research was conducted in the absence of any commercial or financial relationships that could be construed as a potential conflict of interest.

## Abbreviations

ANOVA: one-way analysis of variance
BCA: bicinchoninic acid assay
DMEM: Dulbecco’s modified eagle medium
EDTA: ethylene diamine tetraacetic acid
ESI: electrospray ionization
GTS: ginseng total saponins
HEK: human embryonic kidney
HPLC: high-performance liquid chromatography
OATPs: organic anion-transporting polypeptides
OPD: ophiopogonin D
PBS: phosphate buffered saline
PDG: panaxadiol-type ginsenosides
PTG: panaxatriol-type ginsenosides
SMI: Shenmai injection
TCM: Traditional Chinese Medicine.

## References

[B1] Asl M. N. Hosseinzadeh H. (2008). Review of pharmacological effects of glycyrrhiza sp. And its bioactive compounds. *Phytother. Res.* 22 709–724. 10.1002/ptr.2362 18446848 PMC7167813

[B2] He J. L. Zhou Z. W. Yin J. J. He C. Q. Zhou S. F. Yu Y. (2015). *Schisandra chinensis* regulates drug metabolizing enzymes and drug transporters via activation of nrf2-mediated signaling pathway. *Drug Des. Devel. Ther.* 9 127–146. 10.2147/dddt.s68501 25552902 PMC4277124

[B3] Hu J. Zhang W. Xie Y. M. Wang L. X. Nie X. L. Zhang Y. L. (2012). Meta-analysis of shenmai injection treatment for acute myocardial infarction. *Zhongguo Zhong Yao Za Zhi* 37 2760–2767. 23285929

[B4] Jiang R. Dong J. Li X. Du F. Jia W. Xu F. (2015). Molecular mechanisms governing different pharmacokinetics of ginsenosides and potential for ginsenoside-perpetrated herb-drug interactions on oatp1b3. *Br. J. Pharmacol.* 172 1059–1073. 10.1111/bph.12971 25297453 PMC4314195

[B5] Klatt S. Fromm M. F. Konig J. (2013). The influence of oral antidiabetic drugs on cellular drug uptake mediated by hepatic oatp family members. *Basic Clin. Pharmacol. Toxicol.* 112 244–250. 10.1111/bcpt.12031 23121773

[B6] Kunze A. Huwyler J. Camenisch G. Poller B. (2014). Prediction of organic anion-transporting polypeptide 1b1- and 1b3-mediated hepatic uptake of statins based on transporter protein expression and activity data. *Drug Metab. Dispos.* 42 1514–1521. 10.1124/dmd.114.058412 24989890

[B7] Liu J. Y. Lee K. F. Sze C. W. Tong Y. Tang S. C. Ng T. B. (2013). Intestinal absorption and bioavailability of traditional chinese medicines: a review of recent experimental progress and implication for quality control. *J. Pharm. Pharmacol.* 65 621–633. 10.1111/j.2042-7158.2012.01608.x 23600379

[B8] Lu L. Y. Zheng G. Q. Wang Y. (2014). An overview of systematic reviews of shenmai injection for healthcare. *Evid. Based Complement. Alternat. Med.* 2014:840650. 10.1155/2014/840650 24669229 PMC3942339

[B9] Maeda K. Sugiyama Y. (2010). The use of hepatocytes to investigate drug uptake transporters. *Methods Mol. Biol.* 640 327–353. 10.1007/978-1-60761-688-7_18 20645061

[B10] Mandery K. Balk B. Bujok K. Schmidt I. Fromm M. F. Glaeser H. (2012). Inhibition of hepatic uptake transporters by flavonoids. *Eur. J. Pharm. Sci.* 46 79–85. 10.1016/j.ejps.2012.02.014 22394605

[B11] Marada V. V. Florl S. Kuhne A. Burckhardt G. Hagos Y. (2015). Interaction of human organic anion transporter polypeptides 1b1 and 1b3 with antineoplastic compounds. *Eur. J. Med. Chem.* 92 723–731. 10.1016/j.ejmech.2015.01.011 25618019

[B12] Orazizadeh M. Fakhredini F. Mansouri E. Khorsandi L. (2014). Effect of glycyrrhizic acid on titanium dioxide nanoparticles-induced hepatotoxicity in rats. *Chem. Biol. Interact.* 220 214–221. 10.1016/j.cbi.2014.07.001 25016076

[B13] Sheng J. Tian X. Xu G. Wu Z. Chen C. Wang L. (2015). The hepatobiliary disposition of timosaponin b2 is highly dependent on influx/efflux transporters but not metabolism. *Drug Metab. Dispos.* 43 63–72. 10.1124/dmd.114.059923 25336752

[B14] Tai T. Huang X. Su Y. Ji J. Su Y. Jiang Z. (2014). Glycyrrhizin accelerates the metabolism of triptolide through induction of cyp3a in rats. *J. Ethnopharmacol.* 152 358–363. 10.1016/j.jep.2014.01.026 24486211

[B15] Tang D. (2003). *Science of Chinese Meteria Medica.* Shanghai: Shanghai University of Traditional Chinese Medicine Press.

[B16] Tang S. Li G. Yang R. Xia S. Zang P. Zhang S. (2017). Pharmacokinetics of shengmai injection and shenmai injection in angina pectoris patients. *World Sci. Technol.* 19 825–830. 10.11842/wst.2017.05.019

[B17] Van De Steeg E. Greupink R. Schreurs M. Nooijen I. H. Verhoeckx K. C. Hanemaaijer R. (2013). Drug-drug interactions between rosuvastatin and oral antidiabetic drugs occurring at the level of oatp1b1. *Drug Metab. Dispos.* 41 592–601. 10.1124/dmd.112.049023 23248200

[B18] Wang S. Hu Y. Tan W. Wu X. Chen R. Cao J. (2012). Compatibility art of traditional chinese medicine: from the perspective of herb pairs. *J. Ethnopharmacol.* 143 412–423. 10.1016/j.jep.2012.07.033 22871585

[B19] Xia C. Sun J. G. Hao H. P. Wang G. J. Yan B. Gu S. H. (2008a). Quantitative determination of ophiopogonin d by liquid chromatography/electrospray ionization mass spectrometry and its pharmacokinetics in rat. *Planta Med.* 74 1832–1836. 10.1055/s-0028-1088339 19009500

[B20] Xia C. Wang G. Sun J. Hao H. Xiong Y. Gu S. (2008b). Simultaneous determination of ginsenoside rg1, re, rd, rb1 and ophiopogonin d in rat plasma by liquid chromatography/electrospray ionization mass spectrometric method and its application to pharmacokinetic study of ‘shenmai’ injection. *J. Chromatogr. B Analyt. Technol. Biomed. Life Sci.* 862 72–78. 10.1016/j.jchromb.2007.11.020 18083641

[B21] Xu G. L. Geng D. Xie M. Teng K. Y. Tian Y. X. Liu Z. Z. (2015). Chemical composition, antioxidative and anticancer activities of the essential oil: curcumae rhizoma-sparganii rhizoma, a traditional herb pair. *Molecules* 20 15781–15796. 10.3390/molecules200915781 26343630 PMC6332236

[B22] Ye L. F. Zheng Y. R. Wang L. H. (2015). Effects of shenmai injection and its bioactive components following ischemia/reperfusion in cardiomyocytes. *Exp. Ther. Med.* 10 1348–1354. 10.3892/etm.2015.2662 26622490 PMC4578100

[B23] Yi Y. D. Chang I. M. (2004). An overview of traditional chinese herbal formulae and a proposal of a new code system for expressing the formula titles. *Evid. Based Complement. Alternat. Med.* 1 125–132. 10.1093/ecam/neh019 15480438 PMC516452

[B24] Zeng C. He F. Xia C. Zhang H. Xiong Y. (2013). Identification of the active components in shenmai injection that differentially affect cyp3a4-mediated 1′-hydroxylation and 4-hydroxylation of midazolam. *Drug Metab. Dispos.* 41 785–790. 10.1124/dmd.112.048025 23340957

[B25] Zhan S. Shao Q. Fan X. Li Z. (2015). Development of a sensitive lc-ms/ms method for simultaneous quantification of eleven constituents in rat serum and its application to a pharmacokinetic study of a chinese medicine shengmai injection. *Biomed. Chromatogr.* 29 275–284. 10.1002/bmc.3273 25043947

[B26] Zhang A. Wang C. Liu Q. Meng Q. Peng J. Sun H. (2013). Involvement of organic anion-transporting polypeptides in the hepatic uptake of dioscin in rats and humans. *Drug Metab. Dispos.* 41 994–1003. 10.1124/dmd.112.049452 23396419

[B27] Zhang W. Xiong X. Chen L. Liu M. Xiong Y. Zhang H. (2017). Hepatic uptake mechanism of ophiopogonin d mediated by organic anion transporting polypeptides. *Eur. J. Drug. Metab. Pharmacokinet.* 42 669–676. 10.1007/s13318-016-0384-8 27815731

