# Supplementary material for: Ginsenoside Rb1 and Rd Remarkably Inhibited the Hepatic Uptake of Ophiopogonin D in Shenmai Injection Mediated by OATPs/oatps
Source: Front Pharmacol. 2018 Aug 22;9:957. doi: 10.3389/fphar.2018.00957 (PMC6113708; doi:10.3389/fphar.2018.00957)

**Supplemental Materials**

**Tab. S1. The recoveries of LC-MS method for the determination of ginsenoside Rg1, Re, Rb1 Rd and OPD in SMI**.

|  | | Spiked concentration (ng/mL) | | |
| --- | --- | --- | --- | --- |
|  |  | 10 | 400 | 1500 |
| Rg1 | Recovery (%) | 104.2 | 99.5 | 101.8 |
|  | RSD (%) | 4.65 | 3.33 | 5.69 |
| Re | Recovery (%) | 99.3 | 102.4 | 100.6 |
|  | RSD (%) | 5.96 | 3.28 | 4.49 |
| Rb1 | Recovery (%) | 98.6 | 102.3 | 101.8 |
|  | RSD (%) | 4.88 | 5.12 | 2.86 |
| Rd | Recovery (%) | 99.3 | 103.6 | 101.4 |
|  | RSD (%) | 3.65 | 4.25 | 3.58 |
| OPD | Recovery (%) | 99.5 | 98.8 | 101.2 |
|  | RSD (%) | 5.26 | 3.19 | 4.27 |

The linear range for the determination of ginsenoside Rg1, Re, Rb1 Rd and OPD in SMI were 5-2000 ng/mL. The recoveries were all within 85~115% and the RSD were all less than 15%.

**Tab. S2. The intra- and inter-day accuracy and precision of LC-MS method for the** determination of OPD in rat primary hepatocytes.

|  | | Spiked concentration (μM) | | |
| --- | --- | --- | --- | --- |
|  |  | 0.125 | 0.5 | 2.0 |
| Intra-day | Accuracy (%) | 104.8 | 98.0 | 105.5 |
|  | Precision (%) | 7.63 | 6.12 | 7.58 |
| Inter-day | Accuracy (%) | 98.4 | 104.0 | 98.0 |
|  | Precision (%) | 8.13 | 5.77 | 7.65 |

Good linearity with a correlation coefficient exceeding 0.9998 was observed in the range of 0.03125-4μM and the regression equations for quantification of OPD in rat primary hepatocytes was y=6.5266x-0.1465. The intra- and inter-day precisions expressed as RSD were all less than 15%.

**Tab. S3. The intra- and inter-day accuracy and precision of LC-MS method for the** determination of OPD in the HEK293T cells.

|  | | Spiked concentration (μM) | | |
| --- | --- | --- | --- | --- |
|  |  | 0.03125 | 0.125 | 0.5 |
| Intra-day | Accuracy (%) | 99.2 | 104 | 104 |
|  | Precision (%) | 3.23 | 7.69 | 7.69 |
| Inter-day | Accuracy (%) | 92.4 | 96.0 | 98.0 |
|  | Precision (%) | 12.5 | 7.50 | 6.12 |

Good linearity with a correlation coefficient exceeding 0.9999 was observed in the range of 0.0078125-1μM and the regression equations for quantification of OPD in HEK293T cells was y=6.3235x-0.0605. The intra- and inter-day precisions expressed as RSD were all less than 15%.

**Tab. S4. The intra- and inter-day accuracy and precision of LC-MS/MS method for the** determination of OPD in rat plasma.

|  | | Spiked concentration (ng/mL) | | |
| --- | --- | --- | --- | --- |
|  |  | 7.5 | 30 | 240 |
| Intra-day | Accuracy (%) | 101.2 | 102.83 | 102.17 |
|  | Precision (%) | 4.74 | 8.88 | 3.27 |
| Inter-day | Accuracy (%) | 103.9 | 98.93 | 99.46 |
|  | Precision (%) | 2.18 | 8.15 | 2.43 |

Good linearity with a correlation coefficient exceeding 0.9949 was observed in the range of 2.5-480ng/mLμM and the regression equations for quantification of OPD in rat plasma was y=0.0348x+0.375. The intra- and inter-day precisions expressed as RSD were all less than 15%.

**Fig. S1. The uptake kinetic profile of OPD in OATP1B3-HEK293T cells.**

The whole uptake process of OPD in OATP1B3-HEK293T cells followed a typical Michaelis-Menten equation, and the parameters K_m_ and V_max_ were calculated to be 11.27μM and 146.5 pmol/min/mg protein via GraphPad Prism v5.0, respectively.


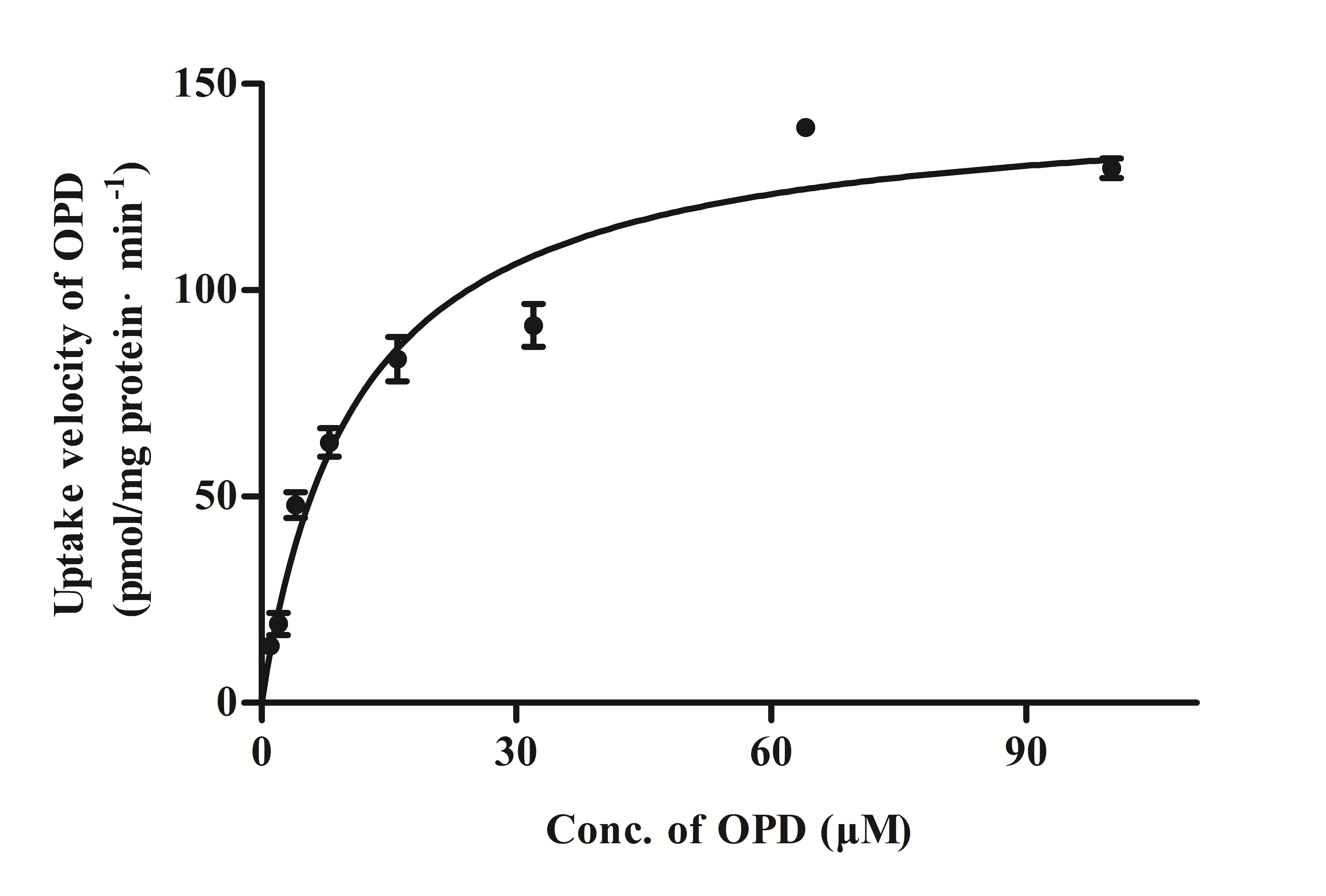


**Fig. S2. The chemical structure of Ophiopogonin D**


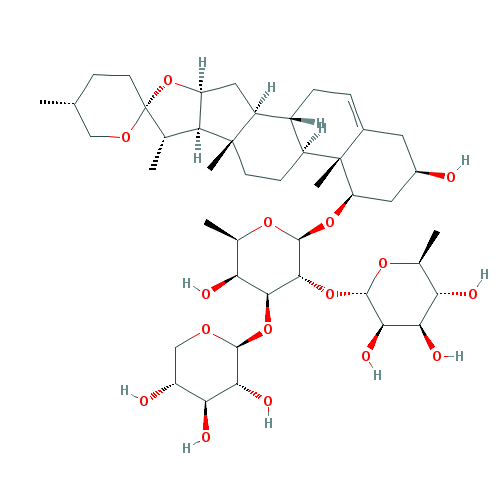

Supplement: Supplementary file 1 [file Table_1.doc]
